# EBNA-1 and VCA-p18 immunoglobulin markers link Epstein-Barr virus immune response and brain’s myelin content to fatigue in a community-dwelling cohort

**DOI:** 10.1016/j.bbih.2024.100896

**Published:** 2024-11-09

**Authors:** Mihály Gayer, Zhi Ming Xu, Flavia Hodel, Martin Preisig, Marie-Pierre F. Strippoli, Peter Vollenweider, Julien Vaucher, Antoine Lutti, Ferath Kherif, Iris-Katharina Penner, Renaud Du Pasquier, Jacques Fellay, Bogdan Draganski

**Affiliations:** a LREN, Centre for Research in Neurosciences, Department of Clinical Neurosciences, Lausanne University Hospital and University of Lausanne, Lausanne, Switzerland; b Neurology Service, Department of Clinical Neurosciences, Lausanne University Hospital and University of Lausanne, Lausanne, Switzerland; c Psychiatric Epidemiology and Psychopathology Research Centre, Department of Psychiatry, Lausanne University Hospital and University of Lausanne, Lausanne, Switzerland; d Division of Internal Medicine, Department of Medicine, Lausanne University Hospital and University of Lausanne, Lausanne, Switzerland; e Division of Internal Medicine, Department of Medicine and Specialties, Fribourg Hospital and University of Fribourg, Fribourg, Switzerland; f Department of Neurology, Inselspital, Bern University Hospital, University of Bern, Bern, Switzerland; g School of Life Sciences, École Polytechnique Fédérale de Lausanne, Lausanne, Switzerland; h Swiss Institute of Bioinformatics, Lausanne, Switzerland; i Precision Medicine Unit, Lausanne University Hospital and University of Lausanne, Lausanne, Switzerland; j Neurology Department, Max-Planck-Institute for Human Cognitive and Brain Sciences, Leipzig, Germany; k University Institute for Diagnostic und Interventional Neuroradiology, Inselspital, Bern University Hospital, University of Bern, Bern, Switzerland

**Keywords:** Epstein-barr virus (EBV), Fatigue, Demyelination, Neuroimaging, Multiple sclerosis (MS), EBV immune response, Population-based cohort study, qMRI, Brain microstructure, Magnetization transfer saturation

## Abstract

Given the association of Epstein-Barr virus (EBV) with subjective perception of fatigue and demyelination in clinical conditions, the question about potential subclinical effects in the adult general population remains open. We investigate the association between individuals’ EBV immune response and perceived fatigue in a community dwelling cohort (n = 864, age 62 ± 10 years old; 49% women) while monitoring brain tissue properties. Fatigue levels are assessed with the established fatigue severity scale, the EBNA-1 and VCA p18 immunoglobulin G (IgG) chronic response – with multiplex serology and the estimates of local brain volume, myelin content, and axonal density - using relaxometry- and multi-shell diffusion-based magnetic resonance imaging (MRI). In our analysis we adjust for the effects of demographic and cardiovascular risk factors, sleep apnea, depression, and polygenic risk score for multiple sclerosis**.** We demonstrate that EBNA-1 IgG levels are positively associated with perceived levels of fatigue, whilst VCA p18 IgG levels show a positive correlation with myelin content and a negative one with an estimate of axonal g-ratio in male participants. In the context of EBVs immune response, the polygenic risk for multiple sclerosis is not associated with increased fatigue levels, brain myelination or atrophy. Our findings bring empirical evidence about the potential role of EBVs chronic immune response in perceived fatigue and hint towards a protective role of myelination specific for men. They underscore the added value of advanced assessment of brain tissue microstructure in uncovering the mechanisms behind frequent fatigue complaints associated with EBV infection and multiple sclerosis.

## Highlights

• *Population-based cohort study tested associations between EBV immune response, fatigue, and brain anatomy.*
• *EBNA-1 IgG are associated with individuals’ fatigue levels.*
• *VCA p18 IgG correlate with higher brain myelin content driven by male participants.*
• *No association between brain myelin differences, MS polygenic risk score and EBV immune response.*
• *EBV immune response may relate to fatigue levels and brain myelin in adulthood.*

## 1 Introduction

There is an ongoing controversy about the role of common viruses, including infection with Epstein-Barr virus (EBV), in the emergence and progression of chronic neuroinflammatory and neurodegeneration (Blackhurst and Funk, 2023). The debate regained attention following studies demonstrating a causal link between an EBV infection and multiple sclerosis (MS) (Bjornevik et al., 2022; Soldan and Lieberman, 2023), similar to reports about the association of EBV reactivation with chronic sequelae of SARS-CoV-2 infection (Couzin and -Frankel, 2022; Rohrhofer et al., 2023). This raises the question about potential subclinical correlates of EBVs chronic immune response on brain and behavior. One main obstacle addressing this question is our limited knowledge about the lifetime trajectories of EBV-induced effects, additionally to their modulation by latent genetic factors.

Greater levels of fatigue, defined as a sense of tiredness, lack of energy or feeling of exhaustion, are reported in both the acute phase of Epstein-Barr virus (EBV) infection (Pedersen et al., 2019; Ruiz-et al., 2021) and in individuals with MS (Braley and Chervin, 2010; Chalah et al., 2015; Zimek et al., 2023; Ayache et al., 2022). Despite the progress in research, defining and measuring fatigue remains challenging (DeLuca, 2024). This motivated the formalization of the fatigue definition as physical tiredness and lack of energy, distinct from sadness or weakness, which is at the core of the proposed Fatigue Severity Scale (FSS)(Krupp et al., 1989). Given the subjective character of fatigue complaints, the assumption of neuroinflammatory response mediated symptoms prevailed in the literature (Ruiz-et al., 2021). Here, the prototypical for neuroinflammatory process demyelination and subsequent neuronal loss are suggested as the common underlying pathophysiological mechanisms (Soldan and Lieberman, 2023; Chalah et al., 2015; Palotai and Guttmann, 2020).

Supporting this notion of a central origin of perceived fatigue, brain imaging studies using diffusion tensor indices from magnetic resonance imaging (MRI) showed microstructural characteristics of damage in fronto-striatal and temporo-insular white matter tracts that were associated with fatigue levels in MS patients (Palotai and Guttmann, 2020). In a broader perspective, the in vivo imaging findings in the debatable nosological entity “chronic fatigue syndrome/myalgic encephalopathy” remain highly controversial demonstrating either opposite brain anatomy correlates or no differences in comparison with healthy controls (Almutairi et al., 2020). One of the possible reasons behind the controversies in the MRI-based computational anatomy literature on the topic is the reliance on non-quantitative data providing arbitrary MR-contrast values. Advanced relaxometry-based MRI offers a window of opportunity to quantify brain tissue properties beyond the assessment of volume or cortical thickness and surface area (Draganski et al., 2011). Further, the biophysical model combining relaxometry-derived magnetization transfer (MT) saturation indicative for myelin content with diffusion-based estimates of axonal density allows for calculating the axon-to-myelin sheath diameter, known from electron microscopy as g-ratio (Stikov et al., 2015). This non-invasive imaging armament is optimally suited for addressing the question about a subclinical EBV immune response-associated gradient of demyelination or axonal loss in the community-dwelling population.

The intricate relationship between the lifelong latency of EBV in humans and the potential consequence of its reactivation remains unclear (Damania et al., 2022). Genome-wide association studies (GWAS) have suggested strong genetic components for developing MS and for the strength of antibody response against EBV infection. Intriguingly, an HLA Class II haplotype (HLA-DR15) has been established as a major genetic risk factor for both traits (International Multiple Sclerosis Genetics Consortium, 2019). Such a finding may be driven by molecular mimicry, where autoantibodies found in MS are also cross-reactive against EBV antigens (Lanz et al., 2022; Tengvall et al., 2019). As such, the chronic presentation of viral antigens could also contribute to auto-immune responses that eventually lead to neuroinflammation (Jelcic et al., 2018). Supporting this hypothesis, a recent study underscored the modulating effects of EBV immune response on the lesion pathology and brain anatomy changes in relapsing remitting MS (Jakimovski et al., 2019). In this Study, the calculation of the individuals’ MS polygenic risk scores allows us to explore subclinical genetic influences related to demyelination, axonal health, and fatigue—traits that could be relevant not only in diagnosed MS but also in individuals with no clinical manifestations of the disease.

Considering the reported causal link between individuals’ immune response to EBV and neuroinflammation, we aimed to address the question whether the magnitude of sustained immune response to EBV, may be associated with the reported fatigue levels and corresponding brain tissue microstructure in the adult community-dwelling population. Considering parametric effects of systemic inflammatory and latent genetic factors, we enrich our analyses with measurements of proinflammatory markers - serum interleukin-1 beta, interleukin-6, tumor necrosis factor-alpha, and a polygenic risk score for MS.

## 2 Materials and methods

### 2.1 Study sample

We analyzed data from CoLaus|PsyCoLaus, a prospective cohort study investigating cardiovascular risk factors and mental disorders in the community-dwelling population of Lausanne (Firmann et al., 2008; Preisig et al., 2009). The BrainLaus cohort (https://www.colaus-psycolaus.ch/professionals/brainlaus/), a subset of CoLaus|PsyCoLaus, provided brain investigation data for 864 participants. None of the participants were diagnosed with MS. The CoLaus|PsyCoLaus study and the BrainLaus cohort were approved by the Institutional Ethics Committee of the University of Lausanne and informed written consent was obtained from all participants (CER-VD, project number PB_2018-00038 (239/09)). In this study we adhered to the relevant STROBE checklist.

### 2.2 Measurements

For fatigue assessment we used the Fatigue Severity Scale (FSS) - a self-reported nine item questionnaire with a 7-point Likert Scale to assess motor fatigue (Galland-Decker et al., 2019). In the study of Krupp et al. fatigue was defined as a sense of physical tiredness and lack of energy, distinct from sadness or weakness. The Fatigue Severity Scale (FSS), based on this definition was initially used in the context of MS fatigue and systemic lupus erythematosus (SLE) (Krupp et al., 1989). While acknowledging its limitations, this assessment has been validated in studies of both MS patients and healthy controls (Lerdal et al., 2005; Valko et al., 2008). For assessment of cardio-vascular profile, we used the SCORE2 (Systematic, COronary Risk Estimation)/SCORE2-OP (for participants with age equal to or larger than 75) model summarising the age-, and country-weighted smoking status, diabetes, systolic blood pressure, total cholesterol level, high-density lipoprotein cholesterol (SCORE2 working group and ESC Cardiovascular risk collaboration, 2021). Sleep difficulties were quantified using the Insomnia Severity Index (ISI) (Bastien et al., 2001) and Berlin score risk for obstructive sleep apnea (OSA) was assessed using the self-reported questionnaire (Netzer et al., 1999), both scores were categorized nominally (0 – no clinical condition, 1 – subthreshold condition, 2- moderate clinical condition, 3 – severe clinical condition). Depression was assessed with the Center for Epidemiologic Studies-Depression 20 item instrument (Carleton et al., 2013) and study participants were categorized (depression: 1, no depression: 0); for details on the depression variable as well as, ISI and Berlin Score for OSA see Galland-Decker et al. (Galland et al., 2019).

### 2.3 Epstein-barr virus serology

From the serum samples, humoral responses to antigens of the infectious agents were analyzed at the German Cancer Research Center, Infections and Cancer Epidemiology Division in Heidelberg (Waterboer et al., 2005, 2006). Seroreactivity was measured at a serum dilution of 1:1000 by using multiplex serology based on glutathione S-transferase fusion capture immunosorbent assays combined with fluorescent bead technology (Hodel et al., 2023); a validation of this method is described in Brenner et al. (2008) (Brenner et al., 2018). Seroreactivity against four EBV antigens (EBNA nuclear antigen-1 (EBNA-1), viral capsid antigen (VCA) p18, Z-Epstein-Barr virus replication activator (ZEBRA) and early antigen-diffuse (EA-D)) was measured using multiplex serology as part of the CoLaus|PsyCoLaus study. For each antigen, individuals were defined as seropositive or seronegative based on the predefined median fluorescence intensity (MFI) thresholds (Hodel et al., 2023). Overall seropositivity against EBV was defined as being seropositive for at least two of the four antigens (Brenner et al., 2018). For the quantitative analysis we focused on VCA p18 and EBNA-1 IgG levels due to their persistence throughout life, and thus validity as markers for strength of humoral immune response even after primary EBV infections (Seigneurin, 2002). EBNA-1 IgG levels are expected to remain high for an extended period lifelong after the time of primary infection and VCA-p18 IgG levels are expected to peak during the acute phase then slowly drop but remain stagnant over time (Seigneurin, 2002).

### 2.4 Brain imaging: relaxometry MRI protocol and quality assessment

All MRI data were acquired on a single 3T system (whole-body Magneton Prisma Siemens Medical Systems, Germany), using a 64-channel coil and following a previously published relaxometry protocol (Trofimova et al., 2021). We ran both automatic and human-led data quality checks to identify and exclude images and participants with abnormally high motion artefacts and abnormalities (Draganski et al., 2011). Exclusion criteria adopted previously (Trofimova et al., 2021) included inter-sequence/intra-sequence artefacts (Castella et al., 2018), macroscopic abnormalities in WM, GM, CSF, and visual inspection with the final number of subjects excluded was 63.

### 2.5 Brain imaging: parameter mapping and brain parcellation

We calculated whole-brain maps of magnetization transfer saturation (MTsat) using the VBQ implementation in the hMRI toolbox (Draganski et al., 2011; Tabelow et al., 2019) and extracted regional values across the cortex and subcortical structures as previously described (Trofimova et al., 2021). The diffusion-weighted imaging (DWI) protocol and subsequent data processing including subject-specific tratography-led data sampling was identical to the reported default settings (Trofimova et al., 2023). Here, we calculate tensor-based indices of fractional anisotropy (FA), mean diffusivity (MD) and neurite orientation dispersion and density imaging (NODDI) estimates of intra-cellular volume fraction (ICVF) to then calculate g-ratio maps (Stikov et al., 2015).

### 2.6 DNA genotyping and polygenic risk score of MS

Study participants were genotyped using the BB2 customised Affymetrix Axiom Biobank array. Genotypes were imputed using the HRC reference panel reference panel and the merged 1000 Genomes Phase 3 - UK10K reference panel (Hodel et al., 2021). We excluded low frequency variants (minor allele frequency <1%), poorly imputed variants (INFO <0.8), and variants that deviate from Hardy-Weinberg equilibrium (P < 10e-17). Approximately 9 million single nucleotide polymorphisms (SNPs) were retained after filtering.

To calculate polygenic risk scores (PRS) for MS, summary statistics from a large-scale GWAS for MS susceptibility was used (International Multiple Sclerosis Genetics Consortium, 2019). PRSice-2 v2.2.7 software was used to calculate PRS based on the “sum” score option (Euesden et al., 2015) on genome-wide significant SNPs (p < 5e-8) to obtain MHC loci specific (MHC PRS), non-MHC loci specific (non-MHC PRS) and global PRS of MS.

### 2.7 Inflammatory markers

For each individual 50 ml venous blood samples were obtained after a night fast, consequently the samples were stored at −80 °C and transported on dry ice. Glucose assays and serum lipids were examined at the Centre Hospitalier Universitaire Vaudois (CHUV) Clinical Laboratory within the timeframe of blood sample collection. Baseline interleukin‐1β (IL‐1β), interleukin‐6 (IL‐6) and tumour necrosis factor‐alpha (TNF‐α) inflammatory markers were measured with multiplex particle‐based flow cytometric cytokine assay (Luminex, lowest detection limit of 0.2 pg/ml). The intra‐ and interassay coefficients of variation (CV) were 15% and 16.7% for IL‐1β, 16.9% and 16.1% for IL‐6 and 12.5% and 13.5% for TNF‐α. Multiplexed particle‐based flow cytometric cytokine assay (Luminex) method was performed at Follow-up, with CV 9.5%. Latex immunoassay (R&D, with interbatch CV 8.0%–7.4% at CoLaus follow‐up and unit of measurement in mg/L) was used to measure high sensitivity C-reactivity protein (hsCRP). Further details on the laboratory assays, measurements and protocol were described in Marques-Vidal et al., 2011) (Marques-Vidal et al., 2011).

### 2.8 Exclusion criteria

Participants were excluded from the study sample if they did not have data in fatigue assessment, brain MRI, DNA genotyping, EBV serology or demographic variables age and sex. Data imputation with the sample median was carried out for the covariates of non interest including SCORE2, insomnia severity index, Berlin score risk for obstructive sleep apnea or depression variable, see number of missing data points in Table 1.

**Table 1:** Characteristics of the Study Sample and the comparison between Study sample and out-of-sample Parent population-based *CoLaus|PsyCoLaus cohort* using two-sample Kolmogorov-Smirnoff statistical distribution test (for continuous variables).

Variables	Study sample (n = 864)	CoLaus|PsyCoLaus (Parent sample) (n = 4881)	Out-of-sample Parent (n = 4017)	KS statistic	p-value KS test
**Sex (% female)**	48.7	55.1	56.4	–	–
**Age (year), mean ± SD**	61.93 ± 9.8	62.93 ± 10.45	63.55 ± 10.63	0.10	<0.001
**Fatigue severity scale score (FSS), mean ± SD**	2.80 ± 1.38	2.88 ± 1.46 (n = 3231)	2.90 ± 1.48 (n = 2367)	0.40	<0.001
**EBV seropositive, %**	96.8	96.8 (n = 3820)	96.8 (n = 2956)	–	–
**EBNA-1 IgG level (MFI), mean ± SD**	5120 ± 3277	5100 ± 3402 (n = 3820)	5095.35 ± 3438 (n = 2956)	0.03	0.59
**VCA p18 IgG level (MFI), mean ± SD**	7333 ± 3426	7573 ± 3404 (n = 3820)	7644 ± 3395 (n = 2956)	0.04	0.18
**CVR: SCORE2 % estimation of CVD event, mean ± SD**	6.65 ± 0.48 (n = 844)	7.56 ± 0.57 (n = 4180)	7.83 ± 0.58 (n = 3336)	0.20	<0.001
**Insomnia severity index (ISI), mean ± SD**	0.22 ± 0.53 (n = 813)	0.24 ± 0.56 (n = 3437)	0.24 ± 0.56 (n = 2624)	0.29	<0.001
**Berlin Score (OSA), mean ± SD**	0.83 ± 0.80 (n = 863)	0.80 ± 0.76 (n = 4647)	0.79 ± 0.75 (n = 3784)	0.06	0.019
**Depression (% depressed)**	48.4 (n = 858)	49.12 (n = 3583)	49.36 (n = 2725)	–	–

Key: EBV: Epstein-Barr virus, CVR: cardio-vascular risk, EBNA-1: Epstein Barr, EBNA-1: EBNA nuclear antigen-1, VCA p18: viral capsid antigen p18, IgG: immunoglobulin G levels, MFI: median fluorescent intensity threshold, CVR: cardiovascular risk SCORE2 percentage, CVD: cardiovascular disorder, ISI: Insomnia Severity Index, OSA: obstructive sleep apnoea Berlin score, SD: standard deviation.

### 2.9 Statistical analysis

We first question the representativeness of the BrainLaus study sample for the CoLaus|PsycoLaus cohort. We compared differences in rate for categorical variables and perform Kolmogorov-Smirnov (KS) two sample distribution test for continuous factors to identify possible selection bias between Study Sample and out-of-sample Parent (consisting of participants in the Parent sample that are not part of the Study Sample). Separately from the KS test we calculated the Cohen’s d effect size to quantify the magnitude of differences in sample means.

Associations between the fatigue levels (dependent variable) and EBV serology markers (VCA p18 and EBNA-1) were established using penalized lasso multiple general linear regression model. We adjusted for the possible confounding effects of age, sex, cardiovascular risk (SCORE2 and SCORE2-OP), sleep difficulties (ISI insomnia severity index), Berlin score risk for obstructive sleep apnoea (OSA) (categorical), depression (categorical), and the polygenic risk scores of MS (global, MHC and non-MHC loci PRS). We also included covariates in the model for the cytokine levels (IL‐1β, IL‐6, TNF‐α and hsCRP). To test for interaction effects we added MS PRS ∗ EBV serology and sex∗EBV serology interaction terms to the model.

In a separate analysis we tested for associations between global brain anatomy characteristics (dependent variable) and EBV serology markers, MS PRS and the EBV serology∗MS PRS interaction terms (independent variables). Distinct models were formulated for the grey matter volume and the myelin indicators MTsat and g-ratio. Confounding effects were adjusted for using covariates of age, sex, cardiovascular risk (SCORE2 and SCORE2-OP) and differences in head size using total intracranial volume or white matter volume (for models with MTsat and g-ratio in the white matter). We included interaction terms in the model to estimate male-female differences in EBV serology markers and MS PRS (sex∗EBV serology, sex∗PRS, sex∗EBV serology∗PRS). Penalized linear regression model was used with Lasso technique for model parameter selection.

In the regional analysis for each brain region, MRI marker, EBV antigen and PRS type, we used a fully automated machine learning model variable selection with LassoLarsIC followed by LassoCV 10-fold cross validation, including the interaction terms between EBV serology and MS PRS (Pedregosa et al., 2011). The MRI markers we tested for in the grey matter regions are mean volume, R2∗ and MTsat. For the white matter tracts we tested for measures of median MTsat, g-ratio, FA, MD and ICVF. In each regional analysis, the p-values of the coefficients were corrected for multiple testing comparison testing using 10′000 permutations, without changing the structure of the covariance matrix, and depending on the test, permuting across brain regions, serology, and MRI metrics (Lindquist and Mejia, 2015).

The input data for each regression analysis was standardised such that the resulting regression coefficients could be used for the estimation of effect sizes. To validate the resulting models, the following statistical information was considered: t statistic, AIC, adjusted R-squared, analysis of residuals, VIF indicator test, model F-test. For data preparation and analysis Python 3.7 was used with packages and version numbers including NumPy 1.20.1, Pandas 1.3.4, Statsmodels 14.0 and Sklearn 1.1.1.

## 3 Results

### 3.1 Demographics, cardio-vascular risk and EBV serology

Information in Table 1 provide an overview of the characteristics of the Study sample (n = 864, women 48.7 percent, age mean ± -SD 61.93 ± 9.8 years), the population-based Parent sample (Colaus | PsyCoLaus) and the out-of-sample (Parent sample excluding Study Sample participants). We compared the distributions of the continuous variables of the Study Sample to those of the out-of-sample Parent sample to assess sample representativeness. The percentage of EBV seropositive participants in the samples correspond and there is no significant statistical difference in the EBNA-1 and VCA p18 IgG levels. The distribution of the age and FSS scores in the study sample differ significantly from that of the out-of-parent sample but the difference in means are small (Cohen’s d effect sizes, 0.15 and 0.07, respectively). The percentage of female participants is lower in the Study sample compared to that of the out-of-sample Parent, this, together with the slightly lower age explains part of the significant differences in the cardiovascular risk scores between samples (SCORE2 results increase with age and are higher for males in general).

### 3.2 EBV serology and fatigue

We tested for differences in EBV immune response associated with levels of fatigue in a parametric analysis. We observed that EBNA-1 IgG levels were positively associated with the fatigue levels (p < 0.05), and the result holds true after correcting for the effects of the potential confounding factors. The estimated effect size from this model can be interpreted such that the increase of EBNA-1 antibodies from sample minimum to seropositivity threshold (MFI (Brenner et al., 2018)) is associated with a 0.1 (95% C.I.: 0.085 0.112) increase perceived score of fatigue (FSS), assuming all other conditions are held constant (Table 2). We found that VCA p18 EBV serology marker were not significantly associated with the fatigue levels. We found no significant associations between the reported fatigue levels and the serum inflammatory cytokines, namely IL‐1β, IL‐6, TNF‐α and hsCRP. These variables were then omitted from the model at the variable selection regularization step. We report that MS PRS and sex∗EBV serology marker interaction terms had no significant association with the fatigue variable and had been omitted during the penalized lasso regression step.

**Table 2:** Multiple linear regression model, estimated standardised beta coefficients with confidence intervals, t-statistics and p-values, measuring the associations between fatigue severity scale score (FSS) and seroreactivity against EBV antigens of EBNA-1 and VCA p18 while correcting for confounding effects.

Dependent variable: Fatigue severity scale (FSS) score Number of observations: 864			Adjusted R-squared: 0.19 F-statistic: 26.3
	**Beta coefficient [CI95%], standardised**	**t statistic**	**p-value**
**Intercept**	0.36 [0.26, 0.46]	7.07	<0.001
**EBNA-1**	0.08 [0.01, 0.15]	2.15	0.031
**VCA p18**	−0.02 [-0.11, 0.08]	−0.32	0.75
**Age**	−0.25 [-0.41, −0.10]	−3.14	0.02
**Sex**	−0.02 [-0.05, −0.01]	−1.13	0.26
**CVR**	−0.06 [-0.22, 0.09]	−0.80	0.42
**Insomnia (ISI)**	0.33 [0.25, 0.42]	7.85	<0.001
**Sleep apnea (OSA, Berlin)**	0.13 [0.08, 0.18]	4.74	<0.001
**Depression**	0.08 [0.06, 0.11]	5.74	<0.001

Key: EBNA-1: EBNA nuclear antigen-1, VCA p18: viral capsid antigen p18, Sex (0: female, 1: male), CVR: cardiovascular risk SCORE2 percentage, ISI: Insomnia Severity Index, OSA: obstructive sleep apnea Berlin score.

### 3.3 EBV serology, MS-PRS and global indices of brain tissue microstructure

We first tested for effects of EBV immune response on the indices of myelin content across the whole brain. We observed a positive association between VCA p18 IgG levels and the MTsat values, (p = 0.0014, details in Table 3A), driven by MTsat differences in the white matter. There were no significant results when testing for associations with individuals’ EBNA-1 IgG levels. We confirm that the effects of MS PRS’s and their interaction with EBV response were not explaining any significant differences in indicators of myelin.

**Table 3A:** General multiple linear regression model, estimated standardised beta coefficients, confidence intervals and p-values, measuring the associations between MT sat signal and EBV serology while correcting for covariates.

Dependent variable: Global MTsat signal		Adjusted R-squared: 0.40 F-statistic: 105.8	
	**Beta coefficient [CI 95%], standardised**	**t statistic**	**p-value**
**Intercept**	0.95 [0.90 0.99]	41.07	<0.001
**EBNA-1**	−0.02 [-0.05 0.01]	−1.22	0.223
**VCA p18**	0.05 [0.01 0.10]	2.46	0.014
**PRS**	0.03 [-0.01 0.07]	1.39	0.166
**Age**	−0.51 [-0.57 -0.44]	−14.95	<0.001
**Sex**	−0.01 [-0.03 -0.01]	−1.37	0.17
**CVR**	−0.14 [-0.20 -0.07]	−4.14	<0.001
**TIV**	0.06 [0.01 0.10]	2.51	0.012

Key: EBNA-1: EBNA nuclear antigen-1, VCA p18: viral capsid antigen p18, sex (0: female, 1: male), CVR: cardiovascular risk SCORE2 percentage, TIV: Total Intracranial Volume.

In the white matter, there was a negative association between the VCA p18 IgG levels and the g-ratio estimates, for men only (sex∗VCA p18 interaction term, Table 3B). The remaining diffusion-derived indices of white matter microstructure did not show significant associations with anti-EBV IgG levels and MS PRS. In the grey matter, there were no significant correlations between the levels of EBV immune response with volume or MTsat.

**Table 3B:** General multiple linear regression model, estimated standardised beta coefficients, confidence intervals and p-values, measuring the associations between g-ratio in the white matter and EBV serology while correcting for covariates.

Dependent variable: g-ratio in the white matter			Adjusted R-squared: 0.38 F-statistics: 95.94
	**Beta coefficient [CI 95%]**	**t statistic**	**p-value**
**Intercept**	0.71 [0.63, 0.80]	17.48	<0.001
**EBNA-1**	−0.01 [-0.05, 0.02]	−0.62	0.538
**VCA p18**	0.02 [-0.05, 0.08]	0.52	0.594
**Sex ∗ VCA p18**	−0.09 [-0.17, −0.01]	−2.22	0.026
**Age**	0.21 [0.14, 0.29]	5.61	<0.001
**Sex**	0.06 [-0.03, 0.09]	3.46	<0.001
**CVR**	0.04 [-0.04, 0.11]	0.97	0.331
**Total WM**	−0.69 [-0.76, −0.61]	−17.61	<0.001

Key: EBNA-1: EBNA nuclear antigen-1, VCA p18: viral capsid antigen p18, sex (0: female, 1: male), sex∗VCA p18 interaction term, CVR: cardiovascular risk SCORE2 percentage, WM: White Matter Volume.

### 3.4 EBV serology, MS-PRS and local white matter microstructure indices

Corresponding to our analysis across the whole brain, our tract-based analysis, we observed a trend for positive association between the VCA p18 IgG levels and the MTsat values across most of the major white matter tracts (not significant after multiple testing correction). The five tracts with the highest measured effect sizes were the thalamo-prefrontal (T_PREF), arcuate fascicle (AF), anterior-thalamic-radiation (ATR), cingulum (CG) and the superior longitudinal fascicles (SLF) I and II (Supplementary Figure 1). The uncorrected results were consistent across these five tracts also for g-ratio metrics associated with VCA p18 IgG levels.

The identical statistical design for associations between anti-EBV IgG levels and the diffusion-based indices of WM microstructure did not show any significant results. There were no significant EBV immune responses – brain anatomy associations in the GM analysis across the cortical and subcortical parcellations. Similarly, the MS-PRS and its interaction with viral antigen indicators did not explain any additional differences in indicators of regional tissue microstructure.

## 4 Discussion

In our representative subsample, taken from a large-scale population-based study, we observed a positive association between individuals’ immune response to EBV - EBNA-1 and the levels of self-perceived fatigue. We report a positive association between the MRI index for myelin content the EBV-specific VCA p18 IgG. Given the causal link between EBV infection and MS, the tests for associations between individual responses to EBV, genetic susceptibility to MS and indicators of brain microstructure pathology did not show significant results. We discuss our findings considering previous reports linking EBV infection with fatigue complaints to then interpret the myelin increases in individuals with stronger immune response to EBV as potential compensatory mechanisms.

Our finding of positive association between the EBNA-1 IgG levels and fatigue scores provides empirical evidence for the potential role of EBV in levels of fatigue in adult life. The observations also corroborate previous reports of increased IgG reactivity against EBNA-1 (Shikova et al., 2020) in patients with chronic fatigue syndrome (CFS). The stage of the viral infection might also be an important factor when comparing the differences in results with respect to the two EBV serology markers. It is yet to be confirmed whether fatigue could be associated with possible reactivation of the virus or a late primary infection.

The current results are different from those presented by Sepulveda et al. Sepúlveda, (2022) reporting no increase in EBNA- and VCA IgG levels of CFS patients, interpreted as deficient EBV-specific B- and T-cell response (Loebel et al., 2014; Ruiz-Pablos et al., 2021). Correspondingly, the finding that individuals’ fatigue levels in the context of EBV immune response are not further modulated by their genetic susceptibility to MS, hints against the assumption of subclinical MS-like fatigue effects triggered by an EBV infection.

The reported positive association between the VCA p18 IgG levels and the index of brain’s myelin content was contrary to the demyelination hypothesis. We interpret the increased myelination as a potential protective mechanism that persists even decades after the acute viral infection. To our knowledge, there are no available longitudinal studies covering the lifespan that could substantiate this assumption. Whether the observed associations measured later in life can be interpreted as protective effects through the viewpoint that these are individuals who did not develop MS is yet to be understood. Aiming to provide a detailed view on potential associations between EBV immune response and white matter microstructure, we leveraged the results of both diffusion weighted imaging and relaxometry-based multi-parameter mapping to obtain a proxy measure of the g-ratio. The finding, that the g-ratio is negatively associated with the EBNA-1 antigen for men only, aligns with the notion of increased level of myelination, a possible post-viral protection observed more so for men than for women. The absence of significant results pertaining to myelin and volume loss in the cortical and subcortical grey matter regions with immune response against EBV and genetic susceptibility to MS is aligned with the white matter findings. These observations enforce the argument against the assumption of sub-clinical brain pathology following EBV infection that might be modulated by individual genetic susceptibility to MS.

We acknowledge some limitations of the present cross-sectional study that does not allow testing causal inferences and longitudinal trajectories. We also do not have the data that would allow for differentiation between primary EBV infection and its reactivation. The correlation between serological data and true viral load in the fatigue is complicated, with conflicting results from previous studies but additional technologies such as peptide microarray or suspension multiplex immunoassay could give further insights (Deary et al., 2018). In future analysis, further variables obtained from GWAS can be included in the modelling to quantify genetic contributions to self-reported tiredness (Deary et al., 2018; Hajdarevic et al., 2022). The limitations are partially mitigated given the opportunity to test for the effects of the interaction between genetic susceptibility to MS and EBV immune response in the context of sub-clinical brain pathology and the availability of behaviour, lifestyle, and demographic data. Another strong argument is that our study is well-powered and utilizes sophisticated brain imaging approaches and high-quality data from a representative subsample of a large-scale population-based study, which enhances the generalizability of the findings to the broader population. Further limitation of the study is that fatigue could be associated with post-viral fatigue syndrome of other viruses than EBV. One possible future extension of this study could be to include data on post-acute sequelae of SARS-CoV-2 infection (Couzin-Frankel, 2022; Rohrhofer et al., 2023).

In summary, the positive association between fatigue levels and EBNA-1 IgG levels supports the potential role of EBV in fatigue. We leveraged both in-vivo quantitative relaxometry based and diffusion MRI data to assess possible signs of brain damage. We found no compelling evidence linking EBV responses and genetic susceptibility to markers, such as demyelination, increased diffusivity, lesions, or volume loss; this finding hints against the notion of potential sub-clinical MS-like damage measured in late adulthood among the general population. Our data revealed a trend of increased myelin content, particularly in men with higher plasma VCA p18 IgG levels. Whether this is a possible compensatory mechanism remains to be understood, and there remains a need for further exploration into the relationship between immune response against EBV and post infection changes in the nervous system on a subclinical level.

## CRediT authorship contribution statement

**Mihály Gayer:** Conceptualization, Formal analysis, Investigation, Methodology, Software, Writing – original draft. **Zhi Ming Xu:** Data curation, Formal analysis, Investigation, Methodology, Software, Writing – review & editing. **Flavia Hodel:** Conceptualization, Data curation, Formal analysis, Software, Writing – review & editing. **Martin Preisig:** Resources, Writing – review & editing. **Marie-Pierre F. Strippoli:** Methodology, Validation, Writing – review & editing. **Peter Vollenweider:** Data curation, Resources, Writing – review & editing. **Julien Vaucher:** Resources, Writing – review & editing. **Antoine Lutti:** Data curation, Funding acquisition, Resources, Software, Writing – review & editing. **Ferath Kherif:** Data curation, Funding acquisition, Resources, Software. **Iris-Katharina Penner:** Methodology, Validation, Writing – review & editing. **Renaud Du Pasquier:** Resources, Writing – review & editing. **Jacques Fellay:** Conceptualization, Funding acquisition, Methodology, Project administration, Resources, Software, Supervision, Validation, Writing – review & editing. **Bogdan Draganski:** Conceptualization, Data curation, Funding acquisition, Investigation, Methodology, Project administration, Resources, Software, Supervision, Writing – original draft, Writing – review & editing.

## Data availability

The data from the CoLaus|PsyCoLaus cohort used in this study cannot be fully shared due to the inclusion of potentially sensitive patient information. According to the competent authority, the Research Ethic Committee of the Canton of Vaud, Switzerland, sharing or transferring this data would violate Swiss legislation designed to protect participants' personal rights. However, non-identifiable, individual-level data can be made available to researchers who meet the criteria for accessing confidential data, for detailed instructions please see https://www.colaus-psycolaus.ch/professionals/how-to-collaborate/) by CoLaus Datacenter (CHUV, Lausanne, Switzerland).

## Sources of funding and support

BD is supported by the Swiss National Science Foundation (project grants Nr. 32003B_135679, 32003B_159780, 324730_192755 and CRSK-3_190185), ERA_NET iSEE project, the Swiss Personalized Health Network SACR project, the Donase and the Leenaards Foundations. LREN is very grateful to the Roger De Spoelberch and Partridge Foundations for their generous financial support. The CoLaus|PsyCoLaus study was supported by unrestricted research grants from GlaxoSmithKline, the Faculty of Biology and Medicine of Lausanne, the Swiss National Science Foundation (grants 3200B0–105993, 3200B0-118308, 33CSCO-122661, 33CS30-139468, 33CS30-148401, 33CS30_177535 and 3247730_204523) and the Swiss Personalized Health Network (grant 2018DRI01).

## Declaration of competing interest

The authors declare the following financial interests/personal relationships which may be considered as potential competing interests: Draganski reports financial support was provided by Swiss National Science Foundation. Draganski reports financial support was provided by European Commission. Fellay reports financial support was provided by Swiss National Science Foundation. Draganski reports equipment, drugs, or supplies was provided by Roger de Spoelberch Foundation. Fellay reports financial support was provided by Swiss State Secretariat for Education Research and Innovation. If there are other authors, they declare that they have no known competing financial interests or personal relationships that could have appeared to influence the work reported in this paper.

## Data Availability

Data will be made available on request.
