# Supplementary material for: EBNA-1 and VCA-p18 immunoglobulin markers link Epstein-Barr virus immune response and brain’s myelin content to fatigue in a community-dwelling cohort
Source: Brain Behav Immun Health. 2024 Nov 9;42:100896. doi: 10.1016/j.bbih.2024.100896 (PMC11609321; doi:10.1016/j.bbih.2024.100896)
Supplement: Multimedia component 1 [file mmc1.docx]

# Supplementary Material


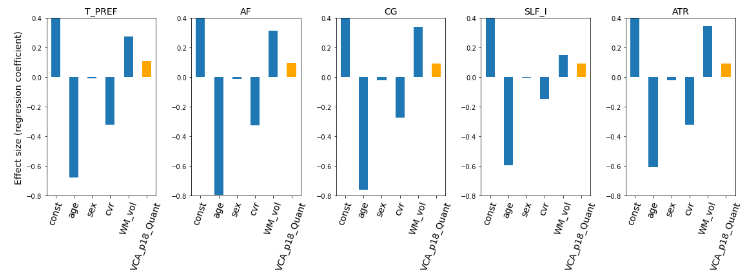


Figure 1. A. *Effect sizes estimated from separate regression model coefficients explaining changes in myelin indicator MTsat. For each of the white matter tracts, from left to right, Thalamo-prefrontal (T_PREF), Arcuate fascicle (AF), Cingulum (CG), Superior Longitudinal fascicle I (SLF_I), Anterior-thalamic radiation (ATR). The values in the regression models were scaled to 0 to 1, abbreviations used: SCORE2 cardiovascular risk (cvr), white matter volume (WM_vol); See the location of the tracts in Fig 3. In the study* [(Wasserthal, Neher, and Maier-Hein 2018)](https://www.zotero.org/google-docs/?Gh2Z4j)*.*

**Equation 1**

$$MTsat{}_{WM}= \beta_{1}* sex+\beta_{2}* age+\beta_{3}* SCORE2 + \beta_{4}*WM volume+{1\beta}_{5} * VCA_{p18}+ {1\beta}_{6} * EBNA1+1 \beta_{7} * PRS +1 \beta_{8} * VCA_{p18}*PRS +... e$$

For example, the case of voxel-based mean of MTsat signal in the cerebral white matter we form the following general multiple regression model, using a base model accounting for the effect of age, sex, differences in cardiovascular health using the SCORE2 aggregate cardiovascular risk indicator and the volume differences in the white matter (or TIV). Further variables are included using penalized regression LASSO only if the variables are tested statistically significant and the resulting model is tested to improve model fit (AIC and adjusted R square tests), shown with indicator function $\boldsymbol{1}$ in the Equation. We tested for polynomial and interaction terms.

**References:**

Wasserthal J, Neher P, Maier-Hein KH. TractSeg - Fast and accurate white matter tract segmentation. Neuroimage. 2018 Dec;183:239-253. doi: 10.1016/j.neuroimage.2018.07.070. Epub 2018 Aug 4. PMID: 30086412.
